# The Good, the Bad, and the Ugly: Mycotoxin Production During Postharvest Decay and Their Influence on Tritrophic Host–Pathogen–Microbe Interactions

**DOI:** 10.3389/fmicb.2021.611881

**Published:** 2021-02-12

**Authors:** Holly P. Bartholomew, Michael Bradshaw, Wayne M. Jurick, Jorge M. Fonseca

**Affiliations:** Food Quality Laboratory, Agricultural Research Service, United States Department of Agriculture, Beltsville, MD, United States

**Keywords:** biocontrol, biofilm, carposphere, metabolite, microbiome, mycotoxin, postharvest pathogen

## Abstract

Mycotoxins are a prevalent problem for stored fruits, grains, and vegetables. Alternariol, aflatoxin, and patulin, produced by *Alternaria* spp., *Aspergillus* spp., and *Penicillium* spp., are the major mycotoxins that negatively affect human and animal health and reduce fruit and produce quality. Control strategies for these toxins are varied, but one method that is increasing in interest is through host microbiome manipulation, mirroring a biocontrol approach. While the majority of mycotoxins and other secondary metabolites (SM) produced by fungi impact host–fungal interactions, there is also an interplay between the various organisms within the host microbiome. In addition to SMs, these interactions involve compounds such as signaling molecules, plant defense and growth hormones, and metabolites produced by both the plants and microbial community. Therefore, studies to understand the impact of the various toxins impacting the beneficial and harmful microorganisms that reside within the microbiome is warranted, and could lead to identification of safe analogs for antimicrobial activity to reduce fruit decay. Additionally, exploring the composition of the microbial carposphere of host plants is likely to shed light on developing a microbial consortium to maintain quality during storage and abate mycotoxin contamination.

## Introduction

There are an estimated 2.2–3.8 million fungal species based on phylogenetic classification and genomic characterizations (Hawksworth and Lücking, 2017). These fungi produce and secrete a diverse array of secondary metabolite (SM) compounds. Among the SM produced are a subset, termed mycotoxins, which are harmful to humans and animals. Although SMs are commonly associated with postharvest and food crop contamination, they are also thought to play important roles in forming and influencing ecological systems (Venkatesh and Keller, 2019). Notably, SMs secreted by phytopathogenic fungi have an intricate relationship with their plant host and biofilm development (Kjeldgaard et al., 2019; Vincent et al., 2020). SM production is influenced by a suite of interactions between the many diverse organisms within the carposphere (collective term for all the microbes that inhabit the fruit surface) (Berg et al., 2016). One area of research that is rapidly gaining traction includes how microbes on the fruit surface, whether intrinsic or applied, interact with each other to influence mycotoxin producing fungi (Dukare et al., 2019; Janakiev et al., 2019; Venkatesh and Keller, 2019). In addition, the production of mycotoxins and other SMs collectively, by phytopathogenic fungi, plays an important role in the development and competition of the organisms within the biofilm matrix (van Rij et al., 2005; Bacon et al., 2006; Martín-Rodríguez et al., 2014).

**FIGURE 1:**
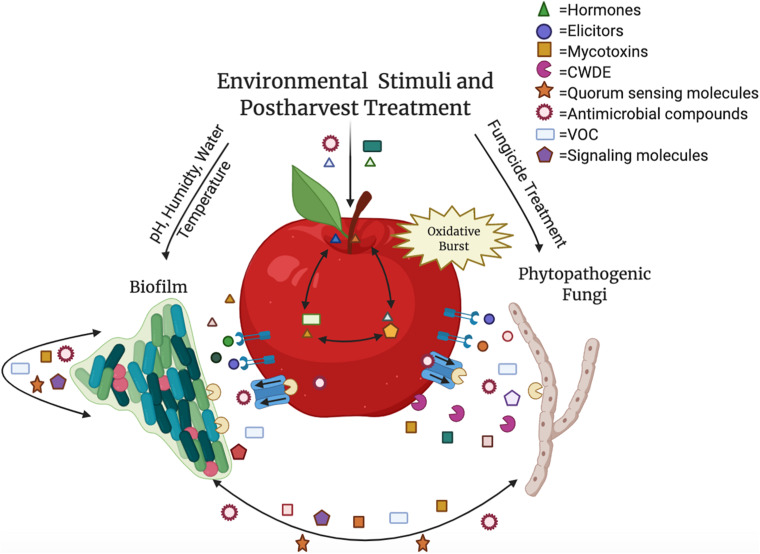
The relationship between the host, pathogen and biofilm is an intricate process containing multiple components. Hormones, volatile organic compounds (VOC), and signaling molecules, such as ethylene, gibberellin, salicylic acid, jasmonic acid, and brassinosteroids, are translocated throughout the fruit as well as produced by microbes and used in postharvest control efforts. Mycotoxins like aflatoxin, patulin, and alternariol are produced by phytopathogenic fungi, and quorum sensing molecules (acyl-homoserine-lactones and autoinducer peptides) are secreted by biofilm-forming microbes. Elicitors are recognized by the fruit in response to pathogens, resulting in an oxidative burst. Both plant and microbial cell wall degrading enzymes (CWDE) and additional antimicrobial compounds are released to manipulate the surrounding species composition. These molecules and abiotic factors during postharvest storage can impact virulence or disease susceptibility, growth, and development of all the interacting organisms within the carposphere. Color variety within molecule categories represents variability within the grouping (e.g., multiple hormones present in the interaction). This figure was produced using Biorender.com.

The tritrophic relationship between the host–pathogen–microbe involves many compounds besides SMs, such as plant hormones, elicitors, cell wall degrading enzymes, quorum sensing molecules, and antimicrobial compounds (Figure 1). The majority of these compounds are involved in microbe–microbe interactions, however, many are SMs that are intricately linked in host (fruit)–biofilm interactions (Jacoby et al., 2020). In addition to playing a role in microbiome communication, these compounds, such as plant hormones, signaling molecules, and volatile organic compounds (VOCs) directly alter the fruit composition and interfere with surrounding microbiota and their SMs (Pusztahelyi et al., 2015; Schulz-Bohm et al., 2017; Quintana-Rodriguez et al., 2018; Jacoby et al., 2020; Vincent et al., 2020). Therefore, understanding how SMs fluctuate in biological interactions and how alterations of SMs impact the host, the microbiome, and the pathogenic microbial composition of the fruit surface can lead to the development of important innovations for the agricultural community.

The goal of this synthesis is to (1) discuss how mycotoxins and SMs from fungi impact host–pathogen–microbe interactions in a postharvest context, and (2) review how SMs and hormones from the fruit and microbes, including those contained in the carposphere, impact each other to provide resistance against phytopathogenic fungi. We aim to combine these different aspects into a comprehensive treatise that enables the complex understanding of carposphere biology and ecology, fungal pathogens, and SMs, and their interplay on fruit during postharvest storage. Understanding the tritrophic dynamics between the host-pathogen-biofilm can lead to efforts to mitigate the negative effects of phytopathogens and to eliminate mycotoxin contamination of stored fruits, grains, and vegetables.

## Secondary Metabolite Production by Postharvest Fungal Pathogens

There are hundreds of SM produced by fungi. They are diverse in form, function, chemical structure, and serve numerous biological and ecological functions. Some of the most notable are mycotoxins, which are defined as SM that are harmful to mammals and humans (Liew and Mohd-Redzwan, 2018). One conservative estimate implicates mycotoxin related losses cost upwards of 5 billion dollars in the United States and Canada alone (Schmale and Munkvold, 2020). Commonly studied sources of mycotoxin outbreaks originate from wheat, cereals, and grains. However, another dominant source is from toxins produced on infected fruits and vegetables during storage. The most well studied mycotoxin-producing fungi from contaminated fruit, that impact human and animal health, include species from the genera *Alternaria*, *Aspergillus*, and *Penicillium* that secrete numerous mycotoxins including, but not limited to, alternariol, aflatoxin, ochratoxin A, citrinin, and patulin (Table 1) (Denning et al., 2006; Solhaug et al., 2016; Alshannaq and Yu, 2017; Escrivá et al., 2017; Zhong et al., 2018; Yu et al., 2020).

**TABLE 1:** Mycotoxins produced by common postharvest phytopathogens.

Phytopathogen	Postharvest fruit association	Common Mycotoxin^a^	Mycotoxin Biosynthesis Clusters	References
*Alternaria alternata*	Pomegranate, pepper, tomato, cherry, peach, citrus (mandarin, tangerine, grapefruit), mango, apple, kiwi, melon, cucumber, fig, pear, litchi, persimmon	Alternariol 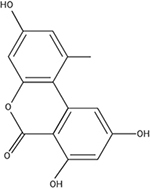	6 genes: *pksI, omtI, moxI, aohR, sdrI, doxI*	Wenderoth et al., 2019; Troncoso-Rojas and Tiznado-Hernández, 2014, Kobiler et al., 2011, Jurick et al., 2014, Ahmad et al., 2020

*Aspergillus flavus*	Grape, peach, fig, tomato, pepper, apple	Aflatoxin B1 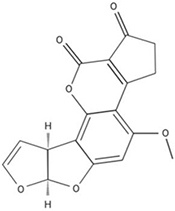	29 genes^b^: *aflF, aflU, aflT, aflC, hypC, aflD, aflA, aflB, aflR, aflS, aflH, aflJ, aflE, aflM, hypE, aflN, hypD, aflG, aflL, hypB, aflI, aflO, aflP, aflQ, aflK, aflV, aflW, aflX, aflY*	Buchanan et al., 1975; Michailides and Thomidis, 2007; Costa et al., 2019; Caceres et al., 2020; Ghuffar et al., 2020

*Aspergillus niger*	Grape, tomato, mango, cherry, banana	Ochratoxin A 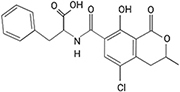	5 genes: *otaA, otaB, otaC, otaD, otaR1*	Wang et al., 2018

*Penicillium citrinum*	Apple, grape	Citrinin 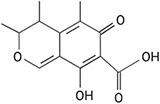	6 genes^c^: *citA (mrl1), citB (mrl2), citC (mrl7), citD (mrl4), citE (mrl6), citS*	He and Cox, 2016

*Penicillium expansum*	Apple, pear, quince, cherry, plum	Patulin 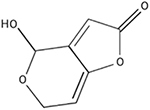	15 genes: *patA, patB, patC, patD, patE, patF, patG, patH, patI, patJ, patK, patL, patM, patN, patO*	Tannous et al., 2014; Ballester et al., 2015; Brito et al., 2020; Jurick et al., 2020

*^*a*^Chemical structure images produced using Biorender.com. Not all mycotoxins produced by these species are represented in this table, and most mycotoxins presented are produced by multiple species.*

*^*b*^Genes presented here are for B-type aflatoxin production.*

*^*c*^The citrinin biosynthesis pathway was described on *Monascus ruber* and is not fully understood in *Penicillium* spp. However, high homology has been found between the species for this biosynthesis cluster (He and Cox, 2016; Schmidt-Heydt et al., 2019).*

*Alternaria* spp. consist of a group of fungal plant pathogens that are the causal agent of fruit rot and produce over 70 different compounds including the detrimental mycotoxin alternariol (Escrivá et al., 2017). Alternariol is a mutagenic, carcinogenic and cytotoxic benzopyrone group mycotoxin that is produced via a chromosomal gene cluster that is transcriptionally regulated by AohR (Wenderoth et al., 2019). Mutagenic effects of host macrophages and formation of micronuclei have also been reported to be caused by alternariol (Solhaug et al., 2016). Despite the harmful effects, alternariol is not regulated by health regulatory agencies such as the FDA in the United States, nor by the European Union. The primary producer of alternariol is *Alternaria alternata*, a causal agent of fruit rot, that has been found apple, cranberry, and pomegranate juices (Jurick et al., 2014; Elhariry et al., 2016; Solhaug et al., 2016). Since *Alternaria alternata* can grow and produce alternariol at low temperatures (Solhaug et al., 2016), storage regimes need to go beyond temperature-controlled environments to prevent outbreaks from occurring via additional changes to storage conditions, pathogen-specific chemical treatments, and/or alteration of the host for resistance.

In addition to alternariol, one of the most toxic mycotoxins produced by *Aspergillus* spp. are from the aflatoxin group. Aflatoxins cause a broad array of diseases loosely categorized as aflatoxicosis. Aflatoxin is problematic to crops growing in the field, especially during drought, but is also prevalent on fruit when stored in humid, warm conditions (Omotayo et al., 2019). Within the U.S., aflatoxin is required to be at concentrations lower than 5 parts per billion (ppb) in milk (specifically aflatoxin M1), 15 ppb in raw peanut, and 20 ppb in other foodstuffs (Food and Drug Administration, 2000, 2019). Some of the most severe impacts of aflatoxins are caused by their ability to bind to proteins non-specifically, cause point mutations within the host DNA, and cause general oxidative damage (Hussein and Brasel, 2001; Caceres et al., 2020). Currently, there are 27 genes encoding enzymes known to be involved in aflatoxin biosynthesis, and these are regulated by AflR and AflS. However, environmental factors such as oxidative stress, pH and nutrient availability also modulate expression (Caceres et al., 2020). Studies inhibiting the production of aflatoxin through regulatory shifts in AflR and AflS have shown that *Aspergillus* cannot infect with the same degree of symptoms, suggesting aflatoxin itself is a causal agent of the symptoms (Yuan et al., 2018, 2019).

Another mycotoxin producer, *Penicillium expansum* is the causal agent of blue mold decay of stored pome and stone fruits. The disease impacts stored apple, peach, and pear, and causes a soft rot that penetrates and macerates the fruit flesh. Patulin, a mycotoxin produced by *Penicillium* spp., *Aspergillus* spp., and *Byssochlamys nivea*, is a common problem for the fruit storage, packing and processing industries. Originally discovered for its antimicrobial effects, patulin was later categorized as a mycotoxin after evidence of its toxicity was found for animals and plants. In humans, patulin causes neurological damage as well as red blood cell death and even gastrointestinal harm (Zhong et al., 2018). Due to the binding affinity to sulfhydryl groups, patulin can inhibit a wide array of enzymes. It also causes oxidative damage to DNA and interferes with numerous host macrophage processes (Puel et al., 2010). In plants, enzymes in ROS detoxification are also impaired (Ismaiel and Papenbrock, 2017). *Penicillium expansum* employs a gene cluster composed of 15 genes involved in patulin secretion, transport and biosynthesis (Tannous et al., 2014; Ballester et al., 2015; Jurick et al., 2020) that enable the ten biochemical steps beginning with conversion of acetyl-CoA and 3 malonyl-CoA into the patulin polyketide compound. Multiple, independent genetic, proteomic and cell imaging data have demonstrated that the final steps in the synthesis occur outside of the fungal cell, as a mechanism to avoid self-toxicity (Li et al., 2019; Jurick et al., 2020). During apple infection, patulin has been found to play an important role in *Penicillium expansum* virulence, as patulin-deficient strains of *P. expansum* were unable to cause wild-type levels of disease symptoms (Sanzani et al., 2012; Snini et al., 2016). Interestingly, patulin production by *P. expansum* is highly variable depending on the apple cultivar (Snini et al., 2016; Kumar et al., 2017). In the U.S., patulin regulations prohibit concentrations exceeding 50 ppb in all apple products, while the EU restricts patulin concentrations beyond 50 μg/kg for juices and ciders, 25 μg/kg in solid foodstuffs, and 10 μg/kg for products intended for consumption by young children. Fermentation can greatly reduce patulin within a given product (e.g., cider), however the mycotoxin is not susceptible to degradation at high temperatures as it is very thermostable (Stinson et al., 1978; Moss and Long, 2002; Omotayo et al., 2019).

Despite the harmful effects commonly associated with mycotoxins, research has shown that mycotoxins and other fungal SMs can act as antibacterial agents. Penicillin, produced by *Penicillium* spp. was the first antibiotic discovered, and targets peptidoglycan production in bacterial cell walls (Gaynes, 2017). *Penicillium* and *Aspergillus* species are producers of the mycotoxin patulin which inhibit bacterial biofilm formation by targeting a transporter for a quorum sensing signaling molecule in *Salmonella* species (Vijayababu et al., 2018). Patulin has also been associated with increasing biofilm formation in a *Bacillus* sp. isolated from dental line water (Liaqat et al., 2008). Similar to *Penicillium* and *Aspergillus*, *Alternaria* spp. also produce antibacterial SMs, such as alternariol 9-methyl ether, altersetin, and macrosporin A (Lou et al., 2016; Escrivá et al., 2017). To date, the effect of these toxins and infectious agents on the carposphere of the fruit host is limited. However, one study in apple showed that fungal infection of *Penicillium expansum* and *Neofabrea* independently resulted in a severe decrease in bacterial colonization in comparison to fungal colonization of the whole apple, demonstrating the broad impact of the toxin producing pathogens on the carposphere during colonization (Wassermann et al., 2019b). Whether the impact was exclusively due to the toxins produced is yet to be determined. These findings suggest a potential for discovery of safe mycotoxin analogs for prevention of undesirable and harmful microbial proliferation, and also provides evidence that mycotoxins could induce population shifts in the existing microbial communities of the fruit surface.

## Bacterial and Fungal Biofilms Impact the Host Microbiome

Secondary metabolites play a central role in microbe-microbe interactions and survival (Spraker et al., 2018; Janakiev et al., 2019). They can cause harm and/or cause alterations to the host organism which can result in differences in nutrient availability, stress compound production, and immunological responses. SMs can also have harmful effects on other microbes within the biofilm by altering colonization requirements and biofilm production and/or direct necrosis.

To understand the interaction of SMs with the bacteria residing within the carposphere of fruit undergoing postharvest decay, it is important to review the lifestyle of bacteria that compose the carposphere. Biofilm formation in bacteria has been extensively studied and many aspects are ubiquitous throughout the diverse prokaryotic group. The process of biofilm formation begins with bacteria attaching to a surface or aggregating with other bacteria, usually due to an environmental cue (e.g., redox compounds, nutrients, low antibiotic concentrations). Using their pili, fimbrae, and sometimes flagella, the bacteria attach and form a microcolony. Often with the help of quorum sensing molecules, these bacteria shift gene expression from their motile and planktonic state to that of a sessile lifestyle for biofilm formation (Li and Tian, 2012). Throughout the biofilm maturation process, the bacteria exude exopolysaccharides and utilize adhesins that help form a matrix between the cells. The inside of a biofilm is quite complex since there can be a rapid reduction in nutrients and oxygen availability, as well as a buildup of many metabolites, quorum sensing signals (i.e., *n*-homoserine lactones, palmitic acid methyl esters), extracellular DNA, and lipids (Yaron and Römling, 2014). Oftentimes, the cells within the interior have a completely different expression profile compared to cells at the exterior of the mature matrix (Kostakioti et al., 2013). The population on the edge of the biofilm will often resume their planktonic lifestyle to disperse and obtain resources for initiating new growth. For bacteria incapable of motility, smaller sessile aggregates will often break off from the mature biofilm that enables migration.

Biofilm formation in fungi has many similarities to that of bacteria in its form, function, and processes (Fanning and Mitchell, 2012; Costa-Orlandi et al., 2017; Mehmood et al., 2019). This is especially true regarding budding yeasts, however, there are fundamental differences between budding yeast and filamentous fungal biofilm formation. Similar to budding yeast, initiation of biofilm formation in filamentous fungi occurs after the planktonic phase when spores or mycelium fragments attach to a surface and begin to reproduce. However, a major difference exists in that filamentous fungi form microcolonies with hyphal growth which then progress to mycelial development, termed mycelial mats, before the final maturation stage (Harding et al., 2009). Once the biofilm is fully formed, dispersal occurs with either sexual spores, conidia, or other fungal propagules (Costa-Orlandi et al., 2017).

For many pathogenic bacteria and fungi, the biofilm serves as a virulence factor (Liu et al., 2016). First, the formation of the biofilm enables microbial protection from host immune responses, as the biofilm itself can trigger host immunity. This protection can extend beyond the immune response to other abiotic stressors, and even antibiotic resistance. One form is through the non-wetting characteristic, due to the thick exopolysaccharide matrix, that excludes liquid penetration (e.g., chemicals, fungicides, etc.) and even some vapors/volatiles (Epstein et al., 2011). Second, biofilms are virulence factors as they can be the direct causal agents of disease symptoms, such as wilt in vascular phytopathogens (i.e., *Ralstonia solanacearum*, *Fusarium oxysporum*). Additionally, biofilms are sometimes formed in monoculture, however, it is more common that there are mixed communities of diverse organisms interacting at the cellular and population levels (Kjeldgaard et al., 2019). These complex structures and influences are described thoroughly by Frey-Klett et al. (2011). Within these mixed communities can be pathogenic microbes, opportunistic pathogens, and beneficial or symbiotic microbes (Droby and Wisniewski, 2018; Janakiev et al., 2019). Therefore, the biofilm often provides a means of protection, nutrient exchange and, regarding pathogenesis, the exchange of pathogenicity islands between surrounding microbes.

The collective consortium of microbial biomass, including these multi-population biofilms, is commonly referred to as the microbiome. Microbiome studies have been at the forefront of microbiology for the last few years (Berg et al., 2014; Compant et al., 2019). The development of the microbiome in plant systems is quite complex and currently not well understood. The rhizosphere microbiome does not necessarily result in phyllosphere similarities due to the vast differences in biotic and abiotic factors, and the carposphere is unique compared to both other microbial niches of the plant. Often, microbial inhabitants of the plant emerge as a seed from the parent plant (Shahzad et al., 2018). After seed emergence, surrounding abiotic and biotic factors such as soil and environmental conditions (i.e., rain and wind), neighboring plants, predators, and transient organisms all come in contact with the plant, each introducing a new batch of microbes (Zarraonaindia et al., 2015; Droby and Wisniewski, 2018; Compant et al., 2019; Kusstatscher et al., 2020). Once harvested, in storage facilities, fruit continue to have exposure to potential biotic agents via storage bins, surrounding fruit, and the air within the facility. In many of these cases, it is challenging to determine whether contact with these microbes will be transient or result in colonization.

Currently there are gaps in the literature that, when investigated, could improve our current understanding of tritrophic interactions. To understand the interactions of these fungal pathogens and their SMs on fruit quality during postharvest storage, an understanding of the core microbiome and their influences on the carposphere composition is critical. While this has been studied for some fruit, including apple, grape, date, tomato, olive, and cucumber, most fruit are understudied (Ottesen et al., 2013; Abdelfattah et al., 2015, 2016; Kecskeméti et al., 2016; Jarvis et al., 2018; Kusstatscher et al., 2020; Piombo et al., 2020). Recent studies found that microbiome composition can be dependent on the fruit structure i.e., peel, stem, surface wound, and calyx of apple or peel and pulp in date (Abdelfattah et al., 2016; Droby and Wisniewski, 2018; Piombo et al., 2020). Additionally, it is likely that different host genotypes result in altered microbiome composition, as well as abiotic factors, and postharvest storage and processing regimes (Martins et al., 2012; Abdelfattah et al., 2016). Evidence of this variability has already been found in apples growing from different rootstocks (Liu et al., 2018) and from apples grown using organic compared to conventional practices (Abdelfattah et al., 2016; Wassermann et al., 2019a). It should be noted that contradictory results were reported in grape, where one study showed shifting of bacterial communities during ripening (Martins et al., 2012) and another revealed the fungal and bacterial microbiome compositions were consistent throughout maturation stages, and across different preharvest regimens (organic, conventional, and biodynamic), (Kecskeméti et al., 2016). Also, some evidence has shown microbial composition shifts are minimal during some postharvest practices, such as hot water treatments and biocontrol agents (BCA) used on stored apples (Wassermann et al., 2019b). These distinct differences reveal that many areas are currently unexplored regarding carposphere fluctuations and establishment of the core microbiome in different fruit systems across different abiotic conditions. Future research evaluating the microbial carposphere composition and the effects of pre and postharvest regimens could ultimately lead to prolonged fruit quality (Buchholz et al., 2018; Linares-Morales et al., 2018).

## Plant Hormones and Signaling Molecules Influence Host Microbial Composition

**FIGURE 2:**
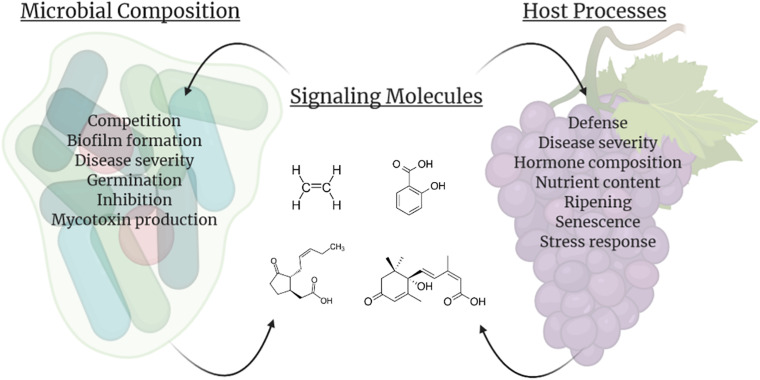
Impacts of signaling molecules (**center**; example molecules listed clockwise are ethylene, salicylic acid, abscisic acid, and jasmonic acid) on the microbial composition associated with the host **(left)** and direct fruit host processes **(right)**. In addition to influencing their own biology and/or populations, producers of these molecules can also impact other species within the vicinity, including facilitating interactions between the host fruit and carposphere. This figure was produced using Biorender.com.

Once in storage many biological processes commence within fruits and vegetables. Senescence and ripening continues, large shifts in fruit chemistry and metabolism occur, and volatile compounds are released. In addition, storage regimens, pathogen exposure, and the microbiome interact to regulate the metabolites and compounds present within this interconnected network on the fruit surface (Figure 2).

The five most common plant growth regulators are auxin (or indole acetic acid; IAA), cytokinin, gibberellin (GA), ethylene, and abscisic acid (ABA). In postharvest storage, these compounds have a key role on ripening, decay, and senescence in the fruit. For example, ethylene is a volatile compound that, when applied to fruit postharvest or accumulated naturally in the environment, induces ripening. The action of ethylene may be manipulated indirectly. Reducing the synthesis of ethylene sensitivity and or production by lowering oxygen availability is commonly used to delay the fruit ripening process given oxygen is required for ethylene production (Paul and Pandey, 2014). Application of ethylene receptor blocking compounds, such as MCP-1, or ethylene biosynthesis disruptor compounds, like hydrogen sulfide, have also been utilized to delay fruit ripening (Schaller and Binder, 2017; Jia et al., 2018; Yao et al., 2020). Ethylene can also be applied to combat postharvest disease. Dong et al. (2020) demonstrated that grapes pretreated with the ethylene stimulator, ethephon, are more resistant to *Botrytis cinerea* infection. Other hormones such as ABA can be used to manipulate ethylene-mediated senescence via postharvest fruit treatment (Luo et al., 2014). Gibberellin can improve shelf life, as it can delay carotenoid changes, maintain firmness, reduce weight loss, and delay ABA degradation (Serrano et al., 2004).

While there are five major plant growth hormones that play a prominent role in the interplay between microbial communities and their host, these are not the only compounds impacting this interaction. Additional hormones and signaling molecules active within the fruit include hormones important for the stress response and defensive signaling such as salicylic acid (SA) and jasmonic acid (JA) (Alkan and Fortes, 2015). SA treatment has benefited the yield of pomegranates during storage, as well as maintained the quality of the harvested fruit (García-Pastor et al., 2020). SA has been shown to stimulate defense-associated phenylpropanoid pathway enzymes when applied to grapes after harvest (Chen et al., 2006). When applied to postharvest citrus fruit via dip treatment, JA and SA were both able to reduce disease severity caused by *Penicillium digitatum* and *P. italicum* through induction of defense enzymes peroxidase and polyphenol oxidase (Moosa et al., 2019). Stimulation of the biosynthesis pathways of these defense hormones has also shown to increase host resistance. For example, the application of chitosan induced JA production in multiple postharvest fruit during *Botrytis cinerea* infection (Peian et al., 2021). Another group of hormones involved in plant growth, brassinosteroids, have been shown to be excellent tools for regulating ripening, as additions of a brassinosteroid, brassinozole, resulted in increased ripening of tomato (postharvest), by altering ethylene and ABA production (Zhu et al., 2015). Similar benefits have been seen for mandarin, eggplant, and mango, where brassinosteroid additions improved nutrient content and reduced chilling injury throughout storage time (Soleimani Aghdam et al., 2016).

These compounds play a vital role in plant health and their synthesis is primarily known to occur within the host fruit. However, recent studies have shown that microbes are able to produce these compounds as well during their interaction with their plant host (Buchholz et al., 2018; Figure 1). Although some of these findings have been attributed to interactions of the microbes with the host phyllosphere in the field, it could be hypothesized that these connections are relevant in postharvest conditions. For example, although *Bacillus amyloliquefaciens* was found to be a useful source of ABA for salt tolerance in rice, it is possible this bacterium could be utilized for postharvest treatment to control ABA availability for fruit maturation in storage (Shahzad et al., 2017). The same could be hypothesized for *Gibberella fujikuroi*, a fungus that produces gibberellin, that causes rice crop to elongate in the field. Many rhizosphere-dwelling and endophytic bacteria can produce IAA and do so when interacting with the plant host (Gamalero and Glick, 2015). Similarly, numerous fungi have been found to produce IAA, including *Aureobasidium pullulans*, *Cladosporium herbarum*, *Epicoccum nigrum*, and *Fusarium* spp. (Frisvad and Thrane, 1993). Clearly, microbial capabilities exist to produce these compounds, and alterations to desired bacterial strains or introductions of the native strain within a postharvest context could enable targeted manipulations of these compounds during storage.

While studies are limited in postharvest conditions, many findings have suggested that the hormones produced by the plant also have effects on microbial colonization, including phytopathogenic fungi. One study showed that cytokinin produced by *Pseudomonas fluorescens* could be used as a BCA for *P. syringae* during *Arabidopsis thaliana* infection, as cytokinin had inhibitory effects on *P. syringae* (Großkinsky et al., 2016). Ethylene was shown to impact germination of multiple fungi, including *Colletotrichum* spp., *Alternaria alternata*, and *Botrytis cinerea* (KępczyńAska, 1989; Flaishman and Kolattukudy, 1994; Kępczyńska, 1994). Furthermore, ethylene was found to block aflatoxin biosynthesis through transcriptional alterations in *Aspergillus* spp. while on peanut (Roze et al., 2004). SA has been shown to negatively impact biofilm in *Pseudomonas aeruginosa*, a bacterial pathogen of humans and plants (Lattab et al., 2017).

Another important group of molecules that are released by the plant, fungi, and bacteria are volatile organic compounds (VOCs). Certain VOCs can induce metabolite release, such as phytoalexins against fungi, and production of VOCs by microbes can in turn induce alterations in the host plant, such as enhanced growth of the plant and/or root systems, or induction of resistance against the microbes (Huang et al., 2012; Schulz-Bohm et al., 2017; Tahir et al., 2017; Quintana-Rodriguez et al., 2018). Some VOCs from the plant provide antimicrobial activity toward bacteria and fungi (Morath et al., 2012; Xu et al., 2019), however, these compounds can sometimes result in phytotoxicity toward the fruit itself (Brilli et al., 2019). Ultimately, the culmination of studies reveals a complicated relationship between the abundance of diverse compounds within the system, and the delicate balance each plays in the vast network of organisms interacting at the carposphere.

## Applications and Translation of Microbiome Data for Improved Product Quality

As 40–50% of postharvest fruit and vegetable waste occurs each year, it is critical to continue striving for methods to maintain fruit quality during the entire postharvest storage process (Alkan and Fortes, 2015; Buchholz et al., 2018). Current practices for food storage include a variety of implemented methods such as a controlled atmosphere, chemical and fungicide treatments for prolonging quality, and coatings and waxes. Choices for mechanical transport and processing, as well as temperature, pressure, and oxygen availability are all taken into consideration during postharvest transport and storage (Villers, 2014). For manipulation of ripening, as well as for pathogen prevention, chemical and biological sprays and fumigation techniques have been implemented. To control mycotoxin production, current methods involve synthetic fungicides, often with single site mode of action targets. However, general concern over chemical residue levels (e.g., Minimum Residue Levels; MRLs) on produce destined to export for other countries has led to a shift in research toward alternative methods.

Biocontrol agents are an increasingly popular way to combat fungal contamination during storage as a safe alternative to fungicidal chemicals and can be derived from microbiome studies. These BCAs can target other live pathogens or biodegrade the toxins produced by those pathogens (Vanhoutte et al., 2016; Leyva Salas et al., 2017). The most successful BCAs currently used on fruits and vegetables are Bio-Save^®^ 100 and 110. They are based on *Pseudomonas syringae* isolate ESC-10 or ESC-11 that is sold in a lyophilized medium. Once the product is hydrated, it is applied pre storage and is effective in reducing several diseases via its rapid growth and colonization of wounds (Janisiewicz, 1988; Bull et al., 1997; Janisiewicz and Korsten, 2002). Bio-Save^®^ targets multiple postharvest pathogens infecting fruit such as *Penicillium expansum*, *Botrytis cinerea*, *Penicillium digitatum*, *Penicillium italicum*, *Mucor piriformis*, and *Geotrichum candidum*, as well as potato rot pathogens (Cerexagri Inc., 2017). Fungi within the same genus can also act as BCAs, as seen with *Aspergillus niger* inhibiting *Aspergillus flavus* from producing aflatoxin in corn (Horn and Wicklow, 1983), or the commercially available BCA Afla-guard^®^ GR, which is a non-pathogenic *A. flavus* strain that is effective in displacing potential pathogenic strains in corn and peanuts (Dorner and Lamb, 2006). To combat *Penicillium* spp. and the patulin they produce, numerous bacterial and fungal species have been employed. The fungi include *Rhodotorula glutinis* (Castoria et al., 2005), *Cryptococcus laurentii* (Tolaini et al., 2010), *Pichia caribicca*, which degrades patulin directly (Cao et al., 2013), and *Candida sake*, which acts as a BCA in cold storage (Morales et al., 2008). The bacteria *Bacillus subtilis*, *Rhodobacter sphaeroides*, *Agrobacterium tumefaciens*, and *Pantoea agglomerans* can be used to reduce *Penicillium* growth and detoxify patulin (Morales et al., 2008; Wang et al., 2016). For combatting harmful *Aspergillus* spp. and aflatoxin production, *Lactobacillus* sp. have been found to bind aflatoxin and metabolize it, thereby reducing overall amounts present (Hamidi et al., 2013), and it is proposed these could be used as an added supplement or probiotic to reduce exposure to aflatoxin. *Streptomyces* spp. reduces aflatoxin production in both *Aspergillus flavus* and *Aspergillus parasiticus* via the antibiotic blasticidin (Yoshinari et al., 2010; Verheecke et al., 2015). A bacterial isolate from soil, identified as a member of the *Stenotrophomonas* genus, has also been found to produce aflatoxin inhibitors (Jermnak et al., 2013). Some microbes are capable of inhibiting a broader array of pathogens, such as the yeast, *Rhodosporidium paludigenum*, which is effective at inhibiting *Alternaria alternata* infection during postharvest storage of Chinese winter jujube fruit (Wang et al., 2009), and *P. digitatum* in citrus fruit (Lu et al., 2013). For broader pathogen control, lactic acid bacteria have been applied within edible biodegradable coatings to prevent harmful fungal and bacterial colonization on numerous postharvest fruit (Linares-Morales et al., 2018; Marín et al., 2019).

## Concluding Perspectives

By no means does this review encompass every aspect of this complex tri-trophic system. Phytoalexins, cell wall degrading enzymes, siderophores, reactive oxygen species, and an abundance of nutrients are but a few additional compounds that impact the interactions discussed here. The breadth of this area of research is broad but critical for understanding the complex dynamic interplay between the host, the carposphere, and the invading pathogens. While some headway has been made for food microbiome studies (Berg et al., 2014; Abdelfattah et al., 2016; Jarvis et al., 2018), there are still large gaps in core microbiome studies and studies involving postharvest crops. Investigating the effects of individual SMs or other compounds from these interactions, including non-toxic SM analogs, would be a more targeted approach to determining the specific impacts they have on the system, and could lead to creative solutions for antimicrobial, preservation, or ripening products for postharvest applications. In conjunction with microbiome studies, metabolomic investigations can reveal the compounds present over the course of these interactions and further shed light on this tritrophic dynamic. Understanding these systems and the influences that shift the microbial consortium is predicted to lead to improved postharvest storage techniques that will reduce food waste, abate mycotoxins and improve fruit quality while being environmentally friendly.

## Author Contributions

HB, WJ, and JF designed the review manuscript. HB, WJ, and MB wrote the manuscript. MB and HB made the figures. All authors contributed substantially in the editing process.

## Disclaimer

All opinions expressed in this manuscript are the author’s and do not necessarily reflect the policies and views of USDA, DOE, or ORAU/ORISE.

## Conflict of Interest

The authors declare that the research was conducted in the absence of any commercial or financial relationships that could be construed as a potential conflict of interest.
